# New Markers of Early Kidney Damage in Children and Adolescents with Simple Obesity

**DOI:** 10.3390/ijms251910769

**Published:** 2024-10-07

**Authors:** Anna Medyńska, Joanna Chrzanowska, Agnieszka Zubkiewicz-Kucharska, Danuta Zwolińska

**Affiliations:** 1 Clinical Department of Paediatric Nephrology, Wroclaw Medical University, 50-367 Wrocław, Poland; danuta.zwolinska@umw.edu.pl; 2 Clinical Department of Paediatrics, Endocrinology, Diabetology and Metabolic Diseases, Wroclaw Medical University, 50-367 Wrocław, Poland; joanna.chrzanowska@umw.edu.pl (J.C.); agnieszka.zubkiewicz-kucharska@umw.edu.pl (A.Z.-K.)

**Keywords:** children, obesity, markers of early kidney damage

## Abstract

The impact of obesity on kidney injury and the development of chronic kidney disease (CKD) is well documented. Unfortunately, the early stages of CKD are asymptomatic, leading to a delayed diagnosis and a worse prognosis. There is a need for more sensitive indicators of kidney damage than those currently used. We aimed to assess the usefulness of serum t-CAF, urinary netrin-1, α-GST, π-GST, calbindin, and calprotectin as biomarkers of early kidney damage in obese children and to investigate the relationship between these indicators and the degree of obesity. A total of 125 simple obese, normoalbuminuric children and 33 non-obese children as controls were selected. Patients were divided into 2 subgroups according to SDS BMI (I: 2 ≤ 4, II: >4). Serum t-CAF was significantly higher in the obese group compared to the controls, as were urinary α-GST, netrin-1, π-GST, and calprotectin. No difference was found between the two obese groups. In normoalbuminuric obese children and adolescents without significant metabolic disorders, serum t-CAF may be a new biomarker for the early detection of renal dysfunction, and urinary netrin-1, α-GST, π-GST, and calprotectin may be better indicators for the detection of early tubular damage, independent of the severity of obesity.

## 1. Introduction

In recent decades, the incidence of obesity and overweight in children and adolescents has increased dramatically, along with comorbidities including kidney disease [1]. Obesity-related kidney disease (ORKD) is the result of the interaction of structural, haemodynamic, and metabolic factors induced by adipose tissue in the kidney. In most cases, ORKDs are asymptomatic and may remain undetected for many years, eventually leading to reduced glomerular filtration and chronic kidney disease (CKD) [2,3,4].

It is estimated that approximately 15–30% of overweight and obese patients will develop CKD [5]. The study, which included 1.2 million adolescents and a median follow-up of 25 years, showed that obese individuals had a 3.4-fold increased risk of developing ESRD from non-diabetic nephropathy [6].

Therefore, it should be extremely important to identify early kidney damage as soon as possible using the most reliable markers.

Unfortunately, most often in everyday practice, the kidney function in children is assessed on the basis of blood creatinine levels and the estimated GFR (eGFR) calculated using the revised Schwartz formula [7]. It is well known that creatinine, due to its numerous limitations, is not a reliable biomarker.

In general, cystatin C is considered a more reliable and specific indicator than creatinine because it is freely filtered by the kidneys, completely reabsorbed, and metabolized in the proximal tubule. Unlike creatinine, serum cystatin C is not influenced by muscle mass or protein intake. However, a threefold increase in cystatin mRNA has recently been demonstrated in adipose tissue of obese adults, suggesting an increased synthesis by adipocytes and a role in adipogenesis. For this reason, cystatin C may not be a good biomarker of kidney function in obese subjects [8]. Microalbuminuria is considered a marker of early kidney damage; already in 2005, Csernus et al. showed its increased values in obese children compared to non-obese children [9].

In obese patients, other biomarkers considered useful in the assessment of early kidney damage, especially in acute kidney injury (AKI), have also been studied. These include NGAL, KIM-1, NAG, and osteopontin as markers of renal tubular damage [10,11]. More recently, serum alpha-1 acid glycoprotein has been shown to be elevated in children with simple obesity before the onset of microalbuminuria, suggesting its usefulness as a marker of early glomerular damage [12].

Recent studies have identified the C-terminal agrin fragment (t-CAF) as a new, more reliable indicator of kidney dysfunction. Agrin is a heparanosulfateproteoglycate (250 kDa) that is highly expressed in the brain and neuromuscular synapses. It is also the major proteoglycan of the glomerular basement membrane, renal tubules, and a component of the extracellular matrix [13]. Neurotrypsin cleaves agrin into two fragments, of which the smaller 22 kDa C-terminal fragment is filtered in the glomerulus and completely reabsorbed in the proximal tubule [14]. Its usefulness as a biomarker of early renal damage was first demonstrated by Steubl et al. in adults with CKD and after kidney transplantation [15,16].

So far, t-CAF has not been studied in obese children, similar to other indicators of tubular damage, such as glutathione S-transferases (GSTs), netrin-1, calbindin, and calprotectin.

GSTs are detoxifying enzymes that are found on renal tubular cells as the izoenzymes α-GST (proximal tubule) and π-GST (distal tubule), among others. Damage to the tubules results in the release of the enzyme into the urine and the presence of isoforms allows differentiation of the site of damage.

An increase in urinary α-GST and π-GST has been found in the early stages of AKI caused by toxic substances [17], in diabetic nephropathy, and in glomerulopathies associated with proteinuria [18,19].

Netrin-1 is a laminin-bound protein that plays a role in the development of the nervous system. It has also been implicated in angiogenesis, endothelial cell migration and proliferation, and in the regulation of inflammatory processes [20,21]. Physiologically, netrin-1 is absent or minimally present in renal tubular epithelial cells, but under the influence of damaging factors, its expression increases in proximal tubular endothelial cells. Netrin-1 is excreted in the urine and is, therefore, useful as an early marker of renal tubular damage [22].

Calbindin, also called calbindin-D28K, is a calcium-binding protein located mainly in the distal tubule and in the initial part of the collecting tubule. Its expression on these tubular cells is increased as a result of damage to them in various clinical situations [23,24]. Blessy et al. observed an eightfold increase in the urinary excretion of calbindin in patients with cisplatin-induced renal injury [25].

Calprotectin—a pro-inflammatory mediator protein—is released by the immune system cells (neutrophils, monocytes, and macrophages) and the collecting duct epithelial cells [26]. Experimental studies have shown that in response to factors that damage the collecting duct epithelial cells, they increase calprotectin release [27]. Clinical studies have shown that urinary calprotectin allows not only early detection of AKI, but also differentiation of its origin (distinction between prerenal and intrinsic AKI). The usefulness of urinary calprotectin as a novel biomarker differentiating structural from functional AKI has been confirmed in other studies [28,29].

The aim of our study was to assess the usefulness of serum t-CAF levels, urinary netrin-1, α-GST, π-GST, calbindin, and calprotectin as a biomarker of early kidney damage in children with simple obesity, and to determine the site of nephron damage. The second aim was to investigate whether there is a relationship between the above markers and the degree of obesity.

## 2. Results

Serum total cholesterol and LDL cholesterol levels did not differ between the obese group and the non-obese subjects, but triglyceride levels were higher and HDL cholesterol lower in obese children compared with non-obese subjects. The serum creatinine was significantly lower in the obese group than in the non-obese subjects, but within the normal range, while eGFR was higher in the obese group. ACR was similar in both groups, with values within normal limits in all children (<30 mg/g creatinine). Fasting glucose was above normal in only four children in the obese group, and an abnormal result was observed in the 120-min glucose challenge of the oral glucose tolerance test. Detailed data are presented in Table 1.

Serum cystatin C and t-CAF were significantly higher in the obese group than in the non-obese subjects. Urinary levels of α-GST, netrin-1, π-GST, and calprotectin were significantly increased in the obese group compared to the non-obese subjects. The urinary calbindin levels were also higher, but without statistical differences. Detailed data are shown in Table 2.

When comparing the above parameters between groups with different degrees of obesity, no significant differences were found (Table 3 and Table 4).

A positive correlation was found between t-CAF and urinary π-GST, netrin-1, and calbindin (r = 0.29, *p* = 0.029; r = 0.27, *p* = 0.045; r = 0.3, *p* = 0.024). Positive correlations were also observed between urinary protein excretion—markers of renal tubular damage—and ACR, as well as between each other (Table 5). A positive correlation was also found between cystatin C and triglyceride (r = 0.19; *p* = 0.042).

## 3. Discussion

Since obesity has been shown to be harmful to the kidneys, there has been increased interest in finding the biomarkers of early kidney damage. Unfortunately, there is still a paucity of studies in children and no consensus on the reliability of different indices of renal function. Recently, microalbuminuria and cystatin have been recognized as reliable biomarkers for the early detection of obesity-related kidney damage [30]. Various markers of tubular damage have also been tested, but the results have been inconclusive [10,11,31].

Our aim was to investigate serum cystatin C, t-CAF, and selected urinary markers of proximal, distal, and collecting tubule damage in obese patients without microalbuminuria and normal serum creatinine levels. It should be noted that cystatin C is responsible for damage not only to the proximal tubule but also to the glomerulus [32].

The present study shows significantly higher serum cystatin C levels in the obese group compared to non-obese controls, confirming its higher sensitivity than creatinine. These results are consistent with those obtained by Polidori et al. [10] and Gayret et al. [31]. The latter also demonstrated a significant increase in urinary NGAL and osteopontin as markers of proximal tubule damage, while KIM-1 and MMP-9 remained unchanged [31]. In a study of 536 children with simple obesity and higher serum cystatin C levels, Marmarinos et al. also showed a correlation between BMI, age and sex (higher values in males) [33], which was not confirmed in our study. On the other hand, Codoner-Franch et al. found no difference in serum cystatin C concentration between obese and non-obese peers [34]. However, they showed that patients with the highest cystatin C levels had the highest burden of cardiometabolic risk factors. Salman et al. confirmed these observations by showing higher cystatin C levels only in obese children with metabolic syndrome. They also found a positive correlation of cystatin C with markers of lipid disorders such as hypercholestrolemia, triglyceridemia, and elevated LDL cholesterol [35]. The above results are in line with ours regarding triglycerides. Oz-Sig et al. made similar observations concerning cystatin C and obesity. By studying three groups of obese patients with diabetes, metabolic syndrome, and simple obesity, they showed the highest cystatin C levels in patients with type 2 diabetes compared to the other groups [36]. This suggests that cystatin C may be a particularly good marker of early kidney damage in obese children with metabolic disorders. However, this view may be partially undermined by the findings of Naour et al. They showed that obese adults with slightly reduced creatinine clearance had significantly higher serum cystatin C levels and a threefold increase in cystatin C mRNA in adipose tissue. This suggests an increased synthesis of this molecule in the adipose tissue of obese patients, which may partly explain the increase in its serum level [8]. This mechanism may also be responsible for the increased serum cystatin C levels observed in other studies [33,35]. The role of cystatin C in obesity is therefore not entirely clear. It appears to be not only a marker of renal function but may also play a role in adipogenesis. The results of the study by Withzel et al. on cystatin C as a marker of kidney damage should also be taken into account [37]. They showed that triglyceridemia *per se* has a significant effect on the serum concentration of cystatin C. Therefore, in this study we decided to investigate t-CAF, which has been shown in selected adult populations to be a more reliable biomarker of renal function than other conventionally used biomarkers [38,39]. It should be emphasized that t-CAF has not been studied in the obese population of either children or adults.

Our study showed for the first time that serum t-CAF levels were significantly higher in obese children than in their lean peers. However, it did not depend on the degree of obesity, suggesting that body fat activity *per se* is not the most important factor. There was also no correlation between t-CAF and cystatin C, creatinine, and eGFR, in contrast to the authors, who studied adults with CKD and kidney transplant recipients [15,16,38,39]. Steubl et al. not only found elevated plasma t-CAF levels in patients with CKD but also showed that it changes faster than creatinine concentration with renal impairment, similar to the decrease in t-CAF after successful transplantation [16]. The above results demonstrate that t-CAF allows early identification of patients at risk of rapid progression to CKD and graft loss. Similar conclusions were drawn by Soliman et al. and Rajeswari M, who showed that plasma t-CAF increases progressively with the degree of CKD [38,39]. They also found that t-CAF discriminated between healthy subjects and patients with stage 1 CKD, confirming its usefulness as a marker of early damage [38]. This statement can also be applied to obese children, as in our study’s population elevated t-CAF levels preceded microalbuminuria. It’s worth mentioning that t-CAF was also found to be a good indicator for early diagnosis of AKI in adult patients with myocardial infarctions [13].

It is likely that the increased t-CAF in the obese population reflects structural changes, both glomerular and tubular [40]. Agrin is an important component of the glomerular basement membrane and renal tubules, which may be damaged in obesity. The positive correlation we have shown between t-CAF and urinary netrin-1, π-GST, and calbindin seems to support this hypothesis. There is probably an abnormal degradation of t-CAF in damaged renal tubules, which, in addition to the reduced glomerular clearance, leads to an increase in its serum concentration.

The aim of our study was also to investigate the usefulness of commonly recognized markers of early damage to different types of renal tubules.

Urinary netrin-1 and α-GST were chosen to assess proximal tubule damage, of which α-GST has never been studied in obese populations, either in children or adults.

We found significantly elevated urinary netrin-1 in the obese children without microalbuminuria compared to the controls, indicating earlier damage to the proximal tubule. We also found that the severity of obesity had no effect on this phenomenon. The concomitant positive correlation between albuminuria and urinary netrin-1 excretion suggests that proteinuria may occur with further tubular damage, as has been shown in diabetic patients [41].

These results are in agreement with those obtained by Övünç Hacıhamdioğlu at al. who studied 68 normoalbuminuric and normotensive obese children and adolescents [22]. In addition, contrary to our observations, they showed higher urinary netrin-1 in children with insulin resistance compared to those without these disorders. This may be related to the small size of the obese group with abnormal glucose metabolisms. It is most likely that the increased carbohydrate metabolism disturbances increase the adverse renal effects of other obesity-related factors, as has been shown in other clinical situations [42].

Urinary α-GST has not been studied in the obese population, neither in adults nor in children. In the present study, urinary excretion of α-GST, similar to netrin-1, was significantly greater compared to non-obese peers and did not depend on BMI. A positive correlation between these two biomarkers raises the suspicion of proximal tubule damage in obese children with normoalbuminuria.

There are very few studies on other biomarkers of proximal tubular injury, which confirm our observations [10,11,31]. The vast majority showed an increase in the excretion of these indices, unlike Gul et al. [11], who found no differences. This could be explained by the very small size of the study group.

We chose π-GST, which is expressed only in the distal tubular epithelial cells, to accurately measure distal tubular damage. For the first time, we were able to show a significant increase in urinary π-GST in obese children compared to their non-obese peers, regardless of the degree of obesity. π-GST is one of the antioxidant enzymes and the increase in its urinary excretion may be a response to the damaging effects of increased oxidative stress associated with obesity [43].

Calbindin, which is an indicator of the damage not only to the distal tubules but also to the collecting duct, did not differ between the study groups.

Perhaps the distal tubular damage factors associated with obesity in this case are too weak to increase its renal expression, as is the case with nephrotoxic chemotherapeutic agents other than cisplatin.

When we examined the urinary excretion of calprotectin as a marker of collecting duct damage, we found a significant increase in obese patients compared to non-obese subjects, regardless of the severity of obesity. There are no data in the literature on this topic in children, and the only study is in adults with type 2 diabetes. Ortega et al., who studied two groups of patients, showed significantly higher calprotectin excretion in obese subjects compared with non-obese subjects (BMI > 30 vs. BMI < 30) [44]. These data show that the excess fat tissue adversely affects all structures of the nephron. We believe that the biomarkers we have selected may be useful for early detection of kidney damage, before microalbuminuria and creatinine elevation occur.

This study has limitations. Firstly, it is a single-center, cross-sectional study, so the number of 125 obese children and adolescents may not be representative of the general population. The number of obese patients with obvious metabolic disorders, which may have influenced the results obtained, was relatively small. This may not have allowed a full insight into the cause of the disorders identified. On the other hand, it shows that even in the absence of significant metabolic disorders, obesity itself is harmful to the kidneys.

## 4. Materials and Methods

A total of 125 obese children and adolescents were included in the study. Only patients with simple obesity, no concomitant chronic diseases and no acute infectious diseases at the time of the study were included. The patients were divided into 2 subgroups according to their BMI.

Patients with secondary obesity associated with genetic syndromes, endocrine disorders, or diseases of the central nervous system were excluded.

The control group consisted of 33 non-obese peers with healthy body weight. Patients in the obese and non-obese subject groups did not differ in gender or age. The mean values of BMI, SD-BMI, and systolic and diastolic blood pressure (SBP and DBP) were significantly higher in the obese group compared to the non-obese subjects.

The characteristics of the obese groups are shown in Table 6 and Table 7.

The study was conducted according to the guidelines of the Declaration of Helsinki and was approved by the Bioethics Committee of the Medical University of Wroclaw (No. KB376/2016). Informed consent was obtained from the legal guardians of the children and from the patients over 16 years of age.

All subjects had their blood pressure, height, and weight measured. Height was measured with a Harpenden Stadiometer to within 0.1 cm. Body weight was measured using an electronic scale (SECA, Hamburg, Germany) with an accuracy of 0.05 kg. BMI was calculated using the following formula BMI = body weight (kg)/[height (m)]^2^. According to the WHO recommendations, obesity is defined as two standard deviations (SD) above the median BMI on the Polish reference percentile charts in relation to sex and age [45].

Blood pressure was measured by the oscillometric method using an Omron 705 IT device (Omron Healthcare CO, Kyoto, Japan). Arterial hypertension was diagnosed according to current European recommendations [46] and on the basis of percentile charts for the Polish population [46].

For each subject, serum levels of the total cholesterol, HDL cholesterol, triglycerides, fasting glucose, cystatin C, creatinine, and t-CAF as a marker of renal function were measured. In addition, the insulin resistance index (HOMA-IR) and a glucose load test were performed in the obese subjects.

In all children, urinary creatinine and albumin were measured, and the albumin to creatinine ratio (ACR) was calculated. Fasting blood samples were taken from the cubital vein in the morning at the time of the scheduled laboratory tests. The blood was centrifuged at 3000 rpm for 15 min. After separation, the serum was frozen at −80 °C until analysis. Serum creatinine, serum lipids, and carbohydrate parameters were measured in the hospital laboratory on the same day.

Urine samples were collected by the clean-catch method from the first morning void (50–150 mL) and then stored at 4 °C for up to 48 h. The urine was then centrifuged at 800× *g* for 30 min. The supernatant was frozen at −80 °C until assayed.

Urine creatinine, albumin, netrin-1, α-GST, π-GST, calbindin, and calprotectin were measured by ELISA (using R&D Systems, Inc. Minneapolis, MN, USA (creatinine) and Wuhan EIAab Science Co., Ltd., Wuhan, China kits (others)). Albuminuria was expressed as milligrams per gram of creatinine (ACR) and α-GST, π-GST, netrin-1, calbindin, and calprotectin per milligram of creatinine.

Serum cystatin C was measured using ELISA kits from R&D Systems, Inc. (Minneapolis, MN, USA) and t-CAF by ELISA kits from Neurotune AG, Schlieren, Switzerland. All tests were performed according to the manufacturer’s instructions.

### Statistical Analysis

Results were expressed as mean (x), median (M), range (min–max), lower and upper quartiles (25–75 Q), and standard deviations (SD) of continuous variables, and the normality of distribution was tested using the Shapiro–Wilk test. The hypothesis of equality of mean parameters in independent groups was tested by the ANOVA analysis of variance, in heterogeneous groups by the non-parametric Mann–Whitney U test, and homogeneity of variance by Bartlett’s test.

For discrete parameters, the frequency of occurrence of traits in groups was analyzed using the two tests with the Yates correction. For selected pairs of parameters, a correlation analysis was performed using Pearson’s and Spearman’s correlation coefficient. A value of *p* ≤ 0.05 was considered statistically significant. A statistical analysis was performed using the EPIINFO Ver. 7.1.1.14 statistical software package.

## 5. Conclusions

In normoalbuminuric obese children and adolescents without significant metabolic disorders, serum t-CAF may be a new biomarker for the early detection of renal dysfunction, and urinary netrin-1, α-GST, π-GST, and calprotectin may be better indicators for the detection of early tubular damage, regardless of the severity of obesity. Further prospective studies are needed to determine the clinical utility of these biomarkers in the early detection of kidney injury in this population.

## Figures and Tables

**Table 1:** Selected biochemical parameters in the study and control groups.

Variable		Normal Weight Group F/M 18/15	Obese Group F/M 68/57	*p*
Total cholesterol [mg/dL]	mean ± SD range (min–max)	164.1 ± 14.8 133–188	179.2 ± 134.9 111–1611	0.537
HDL-cholesterol [mg/dL]	mean ± SD range (min–max)	59 ± 9.6 34–78	42.2 ± 8.4 27–65	# 5.7 × 10^−6^
LDL-cholesterol [mg/dL]	mean ± SD range (min–max)	94.1 ± 14.9 65–121	100.4 ± 24.4 49–184	0.175
Triglycerides [mg/dL]	range (min–max) median quartile (25–75 Q)	57–120 94 74–105	39–469 107 83.5–141	* 0.00216
Creatinine [mg/dL]	mean ± SD range (min–max)	0.736 ± 0.144 0.54–1.19	0.629 ± 0.123 0.37–0.89	# 4.1 × 10^−6^
eGFR [mL/min/1.73 m^2^]	mean ± SD range (min–max)	125 ± 13.2 96.0–160.0	154 ± 25.1 109–235	# 4.3 × 10^−6^
Fasting glucose [mg/dL]	range (min–max) median quartile (25–75 Q)	75–94 88 85–91	56–153 82 77–82	* 0.0118
ACR [mg/g]	range (min–max) median quartile (25–75 Q)	12.86–16.02 15.17 13.42–15.51	12.78–18.03 14.7 12–15.64	* 0.168

* analysis using the non-parametric Mann–Whitney U test. # *p*-value using scientific notation.

**Table 2:** Serum Cystatin C, t-CAF, urine netrin-1, α-GST, π-GST, calbindin, and calprotectin in the study and control groups.

Variable		Normal Weight Group F/M 18/15	Obese Group F/M 68/57	*p*
Serum Cystatin C [ng/mL] n = 158 (33/125)	range (min–max) median quartile (25–75 Q)	257.2–498.9 320.4 276.6–455.8	427.8–848.2 532.4 496.2–729.8	#* 2.6 × 10^−6^
Serum t-CAF [pM] n = 72 (15/57)	range (min–max) median quartile (25–75 Q)	92.2–137 107.1 99.7–117.1	398.3–585.8 482.3 450.7–516.7	#* 1.6 × 10^−6^
Urine netrin-1 [ng/mg]	range (min–max) median quartile (25–75 Q)	2.97–4.57 3.97 3.63–4.27	3.24–35.67 6.56 4.76–7.03	#* 3.1 × 10^−6^
Urine α-GST [mLU/mg]	range (min–max) median quartile (25–75 Q)	16–21.5 20 17.5–20.8	3.6–40 33.8 25.3–36.6	#* 6.3 × 10^−6^
Urine π-GST [ng/mg]	range (min–max) median quartile (25–75 Q)	1.46–2.2 1.99 1.8–2.08	0.54–4.01 3.5 2.34–3.72	#* 4.2 × 10^−6^
Urine calbindin [pg/mg]	mean ± SD range (min–max)	75.7 ± 5.9 57.7–83.4	113.2 ± 126 62.4–1505.5	0.0904
Urine calprotectin [ng/mg]	range (min–max) median quartile (25–75 Q)	32.8–57.3 45.2 39.6–49.8	18.3–111,1 91.6 66.5–98.7	#* 2.4 × 10^−6^

* analysis with the non-parametric Mann–Whitney U test. # *p*-value using scientific notation.

**Table 3:** Selected biochemical parameters according to the SDS BMI values.

Variable		2 ≤ SDS BMI ≤ 4 n = 65 F/M 31/34	SDS BMI > 4 n = 60 F/M 34/26	*p*
Total cholesterol [mg/dL]	mean ± SD range (min–max)	170.5 ± 30.9 121–259	193 ± 208.8 111–1611	0.414
HDL-cholesterol [mg/dL]	mean ± SD range (min–max)	42.8 ± 8.2 27–64	40.1 ± 8.8 27–65	0.111
LDL-cholesterol [mg/dL]	range (min–max) median quartile (25–75 Q)	49–184 101 81–123	58–142 99 82–115	* 0.314
Triglycerides [mg/dL]	mean ± SD range (min–max)	119.2 ± 57.8 52–369	131.8 ± 70.1 39–469	0.312
Fasting glucose [mg/dL]	mean ± SD range (min–max)	82.5 ± 11.9 65–153	82.6 ± 9.1 56–105	0.952
eGFR [ml/min/1.73 m^2^]	mean ± SD range (min–max)	152.9 ± 25.4 109.2–235.0	157.2 ± 26.3 117–223	0.398
ACR [mg/g]	mean ± SD range (min–max)	13.6 ± 2.6 1.89–6.4	13.7 ± 3.2 1.8–18	0.945

* analysis using the non-parametric Mann–Whitney U test.

**Table 4:** Serum Cystatin C, t-CAF, urine netrin-1, α-GST, π-GST, calbindin, and calprotectin according to the SDS BMI values.

Variable		2 ≤ SDS BMI ≤ 4 n = 65 F/M 31/34	SDS BMI > 4 n = 60 F/M 34/26	*p*
Serum Cystatin C [ng/mL] n = 110 (60/50)	mean ± SD range (min–max)	602.4 ± 124.4 427.8–848.2	612.9 ± 123.1 447.1–793.9	0.659
Serum t-CAF [pM] n = 52 (28/24)	range (min–max) median quartile (25–75 Q)	398.3–585.8 475.7 449.4–510.1	424.5–569.8 485 450.7–518	* 0.417
Urine netrin-1 [ng/mg]	mean ± SD range (min–max)	6.51 ± 4.04 3.78–35.67	6.88 ± 5.01 3.24–31.83	0.672
Urine α-GST [mlU/mg]	mean ± SD range (min–max)	30.9 ± 6.7 7.9–39.7	30.5 ± 7.6 3.6–40	0.781
Urine π-GST [ng/mg]	mean ± SD range (min–max)	3.11 ± 0.74 0.72–3.97	3.02 ± 0.85 0.54–4.01	0.548
Urine calbindin [pg/mg]	mean ± SD range (min–max)	126 ± 181.4 67.1–1505.5	102 ± 13.4 62.4–127.5	0.354
Urine calprotectin [ng/mg]	mean ± SD range (min–max)	83 ± 18.2 18.4–108.2	81.8 ± 21.3 18.3–111.1	0.767

* analysis with the non-parametric Mann–Whitney U test.

**Table 5:** Correlations between t-CAF and tubular biomarkers’ damage and correlations of these biomarkers with each other in whole study group.

	r	*p*
serum t-CAF—urine calbindin	0.3	0.024
serum t-CAF—urine π-GST	0.29	0.029
serum t-CAF—urine netrin-1	0.27	0.045
urine calbindin—urine netrin-1	0.67	0.000
urine calbindin—urine α-GST	0.72	0.000
urine calbindin—urine π-GST	0.83	0.000
urine calbindin—urine calprotectin	0.69	0.000
urine calbindin—ACR	0.72	0.000
urine netrin-1—urine α-GST	0.72	0.000
urine netrin-1—urine π-GST	0.78	0.000
urine netrin-1—urine calprotectin	0.73	0.000
urine netrin-1—ACR	0.74	0.000
urine α-GST—urine π-GST	0.88	0.000
urine α-GST—urine calprotectin	0.88	0.000
urine α-GST—ACR	0.92	0.000
urine π-GST—urine calprotectin	0.88	0.000
urine π-GST—ACR	0.88	0.000
urine calprotectin—ACR	0.87	0.000

**Table 6:** Characteristics of the study and control groups.

Variable		Normal Weight Group F/M 18/15	Obese Group F/M 68/57	*p*
Age [years]	mean ± SD range (min–max)	12.9 ± 3.0 7.6–17.8	13.7 ± 2.84 8.0–17.9	0.172
Body weight [kg]	range (min–max) median quartile (25–75 Q)	47.7 ± 11.9 48.1 38.6–55.4	85.7 ± 23.8 82.7 72.7–100	#* 6.3 × 10^−6^
BMI	range (min–max) median quartile (25–75 Q)	19.2 ± 2.3 19 17.7–20.3	32.1 ± 5.8 30.8 28.4–35.2	#* 7.1 × 10^−6^
SDS BMI	range (min–max) median quartile (25–75 Q)	0.061 ± 0.633 0.061 (−0.547)–0.563	4.02 ± 1.7 3.55 2.88–5.13	#* 5.3 × 10^−6^
SBP [mmHg]	mean ± SD range (min–max)	106.3 ± 8.9 85–120	117.2 ± 9.9 98–140	# 1.4 × 10^−6^
DBP [mmHg]	mean ± SD range (min–max)	65.3 ± 7.2 48–76	71.8 ± 8.0 50–92	# 5.1 × 10^−6^

* analysis with the non-parametric Mann–Whitney U test. # *p*-value using scientific notation.

**Table 7:** Patients’ characteristics according to the SDS BMI values.

Variable		2 ≤ SDS BMI ≤ 4 n = 65 F/M 31/34	SDS BMI > 4 n = 60 F/M 34/26	*p*
Age [years]	mean ± SD range (min–max)	13.7 ± 2.8 8–17.9	13.5 ± 2.9 8.2–17.8	0.729
Body weight [kg]	range (min–max) median quartile (25–75 Q)	78.2 ± 17.1 78.2 70.5–88.4	98.6 ± 26.7 99.3 79.9–113.3	#* 3.8 × 10^−6^
SBP [mmHg]	mean ± SD range (min–max)	116.1 ± 9.4 98–140	119.3 ± 10.4 99–140	0.0959
DBP [mmHg]	mean ± SD range (min–max)	70.3 ± 7.7 50–90	73.9 ± 7.9 59–92	0.0172

* analysis using the non-parametric Mann–Whitney U test. # *p*-value using scientific notation.

## Data Availability

Data are contained within the article.
